# Topochemical synthesis of ternary metal sulfides including new LaAgS_2_

**DOI:** 10.1039/d6dt02059c

**Published:** 2026-09-30

**Authors:** Glen R. Hebberd, Toby J. Blundell, Leon Bowen, Stewart J. Clark, Emma E. McCabe

**Affiliations:** a Department of Physics, Durham University South Road Durham DH1 3LE UK emma.mccabe@durham.ac.uk; b Department of Chemistry, Durham University South Road Durham DH1 3LE UK

## Abstract

Ternary sulfides are increasingly sought after for applications as battery materials, photovoltaics and photocatalysts as a result of their small band gaps and good electrical conductivities but their synthesis is often more challenging than that of oxides. We report here the low temperature synthesis of new sulfide LaAgS_2_ by an anionic redox topochemical reaction, alongside the synthesis of LaCuS_2_ by mechanochemical topochemical reaction. Our computational work indicates that reaction enthalpies to form LaAgS_2_, with structure closely related to that of LaCuS_2_, are relatively small, consistent with our synthetic work. Structural analysis suggests this is due to the larger size of the Ag^+^ ion compared with Cu^+^. Diffuse reflectance measurements indicate that both sulfides have band gaps ∼2.1(2) eV.

## Abstract 2

The topochemical synthesis and crystal structure of the new ternary chalcogenide LaAgS_2_, a semiconductor with band gap close to 2 eV.

## Graphical abstract

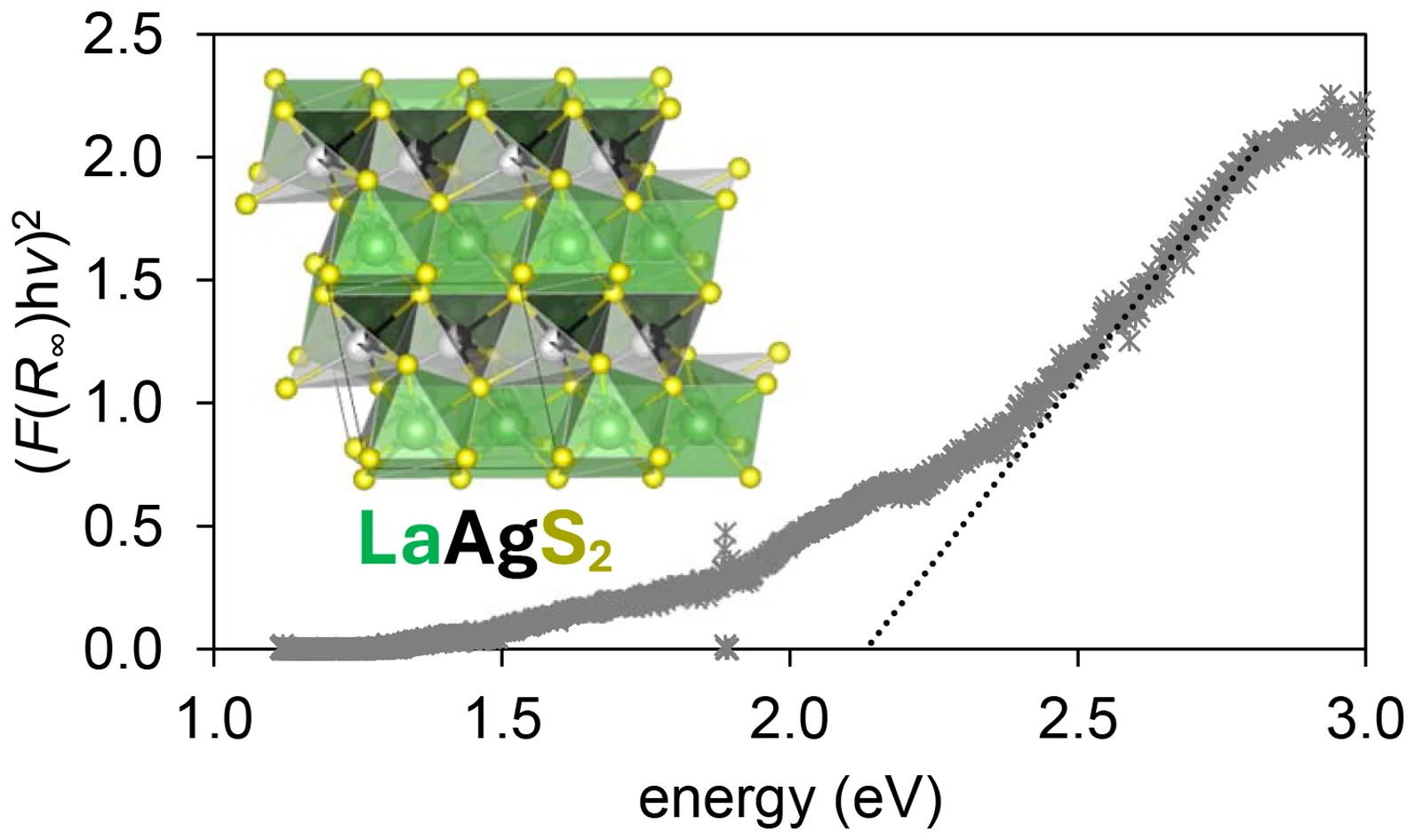

## Introduction

Metal sulfides, often with smaller band gaps and higher electrical conductivities than their oxide analogues, are increasingly sought after for applications in energy storage and conversion, for example as electrodes^1^ or electrolytes in batteries,^2,3^ in supercapacitors,^1,4^ as photovoltaics^5,6^ and as photocatalysts.^7–10^ Their synthesis is therefore very topical^10–12^ and low temperature synthesis routes, which are likely to have lower energy costs and give products with smaller particle sizes,^1,2,7^ are particularly important.

Sasaki *et al.* have demonstrated that topochemical reactions involving anionic redox provide an exciting low-temperature route to synthesise both thermodynamically stable phases,^13,14^ as well as metastable phases.^15^ Examples include the oxidation and insertion of Cu into La_2_O_2_S_2_ to give the transparent p-type semiconductor LaCuOS.^13,14^ (LaCuOS is typically prepared by solid state reaction of oxide and sulfide reagents at temperatures ≥1000 °C,^16,17^ but interestingly, it was first prepared by oxidation of LaCuS_2_.^18^) The oxysulfide precursor La_2_O_2_S_2_ is composed of (001) fluorite-like [La_2_O_2_]^2+^ layers separated by layers of (S_2_)^2−^ dimers. It is these (S_2_)^2−^ dimers that are reduced by the metal reactant species which cleaves the S–S bonds to form a metal sulfide layer.^13,14^ The low temperature at which these reactions can be carried out means that structural rearrangements of the (S_2_)^2−^ – containing precursor are limited, constraining the product to retain some structural features of the precursor. These topochemical anionic redox reactions can also proceed *via* de-intercalation of S^2−^, *e.g.* La_2_O_2_S_2_ + 2*A* → La_2_O_2_S + *A*_2_S (*A* = Na, K, Rb). Importantly, the oA-polymorph of La_2_O_2_S formed by this topochemical reaction retains the (001) fluorite-like [La_2_O_2_]^2+^ layers of the precursor and is metastable to the hexagonal hP thermodynamically-stable polymorph.^19,20^ (oA and hP refer to A-centred orthorhombic and primitive hexagonal unit cells, respectively, using Pearson notation).

In addition to the oxychalcogenides *Ln*_2_O_2_*Q*_2_ (*Q* = S, Se), binary chalcogenides such as BaS_2_ and BaS_3_ have also been shown to undergo topochemical anionic redox reactions.^21^ LaS_2_, containing (S_2_)^2−^ dimers, is also an ideal precursor from which to carry out anionic-redox topochemical reactions to target La*M*S_2_ phases (*M* = monovalent cation). Its structure is composed of rocksalt-like La^3+^ S^2−^ blocks separated by layers of (S_2_)^2−^ dimers. The stacking sequence and the orientation of the (S_2_)^2−^ dimers gives rise to a variety of polymorphs (Fig. 1) including monoclinic (*P*2_1_/*c*) α-LaS_2_ which may be the kinetic product, and orthorhombic (*Pnma* or *Pn*2_1_*a*) β-LaS_2_ which is thought to be more thermodynamically stable.^22–26^

**Fig. 1:**
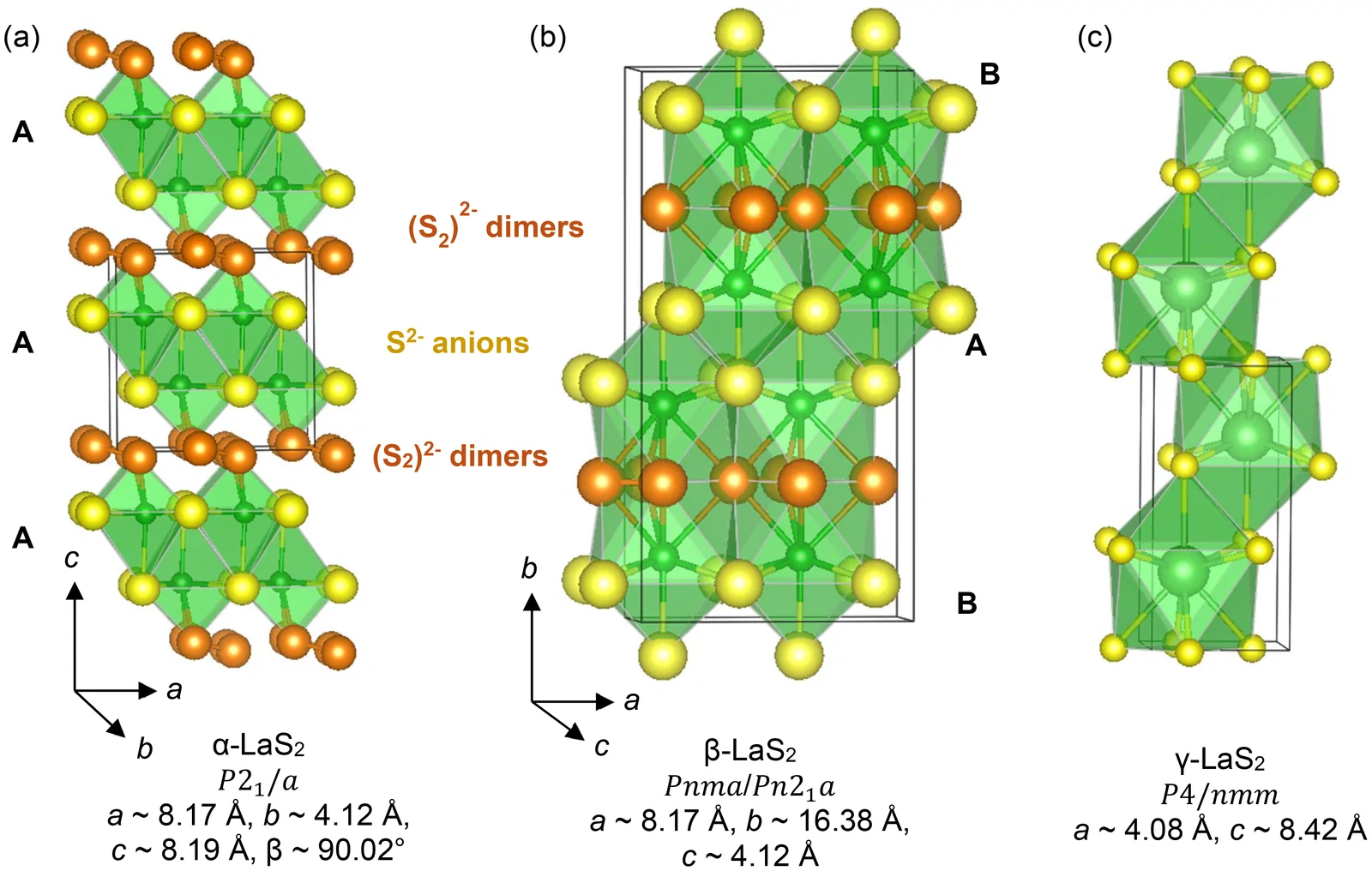
Illustration of the crystal structures for (a) α-LaS_2_, (b) β-LaS_2_ and (c) γ-LaS_2_ which does not have obvious (S_2_)^2−^ dimers. La^3+^, S^2−^ and (S_2_)^2−^ sites are shown in green, yellow and orange, respectively. The stacking sequences AAA and ABA are highlighted for the α and β polymorphs.

The ternary lanthanide copper sulfides *Ln*CuS_2_ are p-type semiconductors with band gaps in the range 2.2–2.5 eV.^27,28^ Several phases including SmCuS_2_ and YbCuS_2_ show interesting magnetic behaviour at low temperatures.^29–31^ A variety of structures have been reported for this series, all comprising CuS_4_ tetrahedra, joined by edges, corners, or both, with *Ln* coordination number decreasing from 7 for larger lanthanides, to 6 for the smaller lanthanides.^32–35^ The main *Ln*CuS_2_ structures are summarised in the SI. These materials are typically prepared by high temperature (*T* ≥ 800 °C) solid state reactions^27,34,36–39^ and we describe here new low-temperature synthesis routes to prepare LaCuS_2_ including low-temperature topochemical reactions as well as analogous topochemical reactions carried out by ball milling (“mechanochemical topochemical” or MT reactions).

This success led us to investigate the silver analogue LaAgS_2_. Several *Ln*AgS_2_ phases (*Ln* = Sm–Yb) are known,^40–42^ and reports of their photocatalytic activity^43^ will no doubt lead to further investigation of possible synthetic routes.^44^ LaAgS_2_, containing the largest lanthanide ion, has not been reported. The structures of *Ln*AgS_2_ phases are quite different from the *Ln*CuS_2_ analogues, with the larger Ag^+^ cation sitting in 5- or 6-coordinate sites.^40–42^ We report here the successful preparation of LaAgS_2_ with structure closely related to that of LaCuS_2_, as a result of the low-temperature topochemical synthesis method.

## Methods

### Synthetic procedures

Precursors Cu_2_S and Ag_2_S were synthesised by solid state reaction of stoichiometric amounts of Cu, Ag (Alfa Aesar, APS 1.3–3.2 micron, 99.9%), and S (Alfa Aesar, −325 mesh, ≥99.5%) at reaction temperatures of 600 °C and 250 °C, respectively, in evacuated, sealed quartz ampoules for 24 hours (see SI).

High temperature synthesis reactions (HT) were carried out by grinding together stoichiometric quantities of La_2_S_3_ (Strem Chemicals, 99.9%-La), S (Alfa Aesar, −325 mesh, ≥99.5%), Cu_2_S, or Ag_2_S as appropriate. All preparation and grinding steps were carried out in an N_2_-filled glovebox with sample mixes placed in alumina crucibles in quartz ampules which were evacuated to <10^−2^ bar, and flame sealed. The reaction ampoules were then heated at 2 °C min^−1^ to 400 °C, 0.5 °C min^−1^ to 600 °C, then 1 °C min^−1^ to the reaction temperature for the high temperature (HT) synthesis route.^22,23^ HT reaction of La_2_S_3_ and S to form LaS_2_ was carried out at 750–820° C for up to 8 days as reported previously.^22,23^ The HT reaction of La_2_S_3_ and Cu_2_S to form LaCuS_2_ was carried out at 900 °C for 3 days. The HT reaction of La_2_S_3_ and Ag_2_S to form LaAgS_2_ was attempted at 800 °C for 3 days.

Low temperature topochemical (LTT) reactions were also carried out from pre-synthesised LaS_2_ with Cu or Ag metal in evacuated, sealed quartz ampoules: LTT reaction of LaS_2_ with Cu to form LaCuS_2_ was carried out at 400 °C for 2 × 24 hours with grinding between heatings; LTT reaction of LaS_2_ with Ag to form LaAgS_2_ was carried out at 300–500 °C for 24 hours.

Mechanochemical topochemical (MT) reactions were also explored, using a Fritsch Pulverisette 6 planetary ball mill fitted with an 80 ml ZrO_2_ milling jar loaded with 24 × 10 mm ZrO_2_ milling balls. The reactant powders were placed into the milling jar under an N_2_ atmosphere, then clamped tightly shut. A total powder mass in the range of 0.7–2.0 g was sufficient for the ball milled reactions and subsequent characterisation steps (giving ball-to-powder ratios between 35 and 105). These mechanochemical reactions were undertaken at 550 rpm for one hour (composed of 5–10 min of milling time followed by 2–3 min pauses). Synthesis of LaS_2_ by this MT method was carried out with 10 mol% excess of sulfur to account for volatilisation.

Reactions were monitored using X-ray powder diffraction (XRPD), with data collected using a Rigaku Miniflex with Cu Ka radiation (*λ* = 1.5418 Å, 40 kV, 15 mA) and a D/teX Ultra detector. Rietveld refinements^45^ were carried out using the TopasAcademic software.^46,47^ Energy-dispersive X-ray (EDX) measurements were made using a Jeol 2100F transmission electron microscope operating at 200 keV. The sample was deposited onto a holey carbon grid. This was mounted in a double-tilt sample holder. EDX data were collected with an Oxford Instruments X-Max 65T silicon drift detector.

### Crystal growth

Twinned single crystals of LaAgS_2_ were grown from the LaAgS_2_ powder prepared by LTT reactions in a CsCl flux (a 1 : 1 molar ratio) within an evacuated, sealed quartz ampule, cooling from 800 °C at a rate of 2 °C hour^−1^. The flux was subsequently removed by washing with deionised water. Crystals were viewed under an optical microscope fitted with a cross-polariser to identify specimens suitable for single crystal X-ray analysis. The X-ray single crystal data for LaAgS_2_ were collected at 120.0(2) K using Mo Kα radiation (*λ* = 0.71073 Å) on a Bruker D8Venture diffractometer with a Photon III MM C7 CPAD detector, IμS-III-microsource, focusing mirrors equipped with a Cryostream (Oxford Cryosystems 700+) open-flow nitrogen cryostat. The structure was solved using Olex2^48^ with the ShelXT^49^ structure solution program using Intrinsic Phasing and refined with Olex2.refine^50^ refinement package using Least Squares minimization on *F*^2^. All atoms were refined with anisotropic atomic displacement parameters. Crystal data and parameters of refinement are listed in Table 1. Crystallographic data for the structure have been deposited with the Cambridge Crystallographic Data Centre with deposition number CCDC 2580779.

Details from analysis of single crystal XRD data for LaAgS_2_. Refined parameters (including anisotropic atomic displacement parameters) and selected bond lengths are given below

**Table:** 

Chemical formula	LaAgS_2_
Formula weight (g mol^−1^)	310.906
Crystal system	Monoclinic
Space group	*P*2_1_/*c*
*a* (Å)	6.9932(7)
*b* (Å)	7.3224(8)
*c* (Å)	7.1599(7)
*β* (°)	102.225(4)
Volume (Å^3^)	358.32(6)
*Z*	4
*D*_calc_ (g cm^−3^)	5.763
Radiation (Å)	Mo Kα 0.71073
Temperature (K)	120.0(2)
Crystal size (mm)	0.116 × 0.069 × 0.017
*μ* (mm^−1^)	18.047
2*θ* max (°)	30.21
*F*(000)	541.363
Reflections collected	8642
Unique reflections	1070 [*R*_int_ = 0.0610, *R*_sigma_ = 0.0348]
	
Data/restraints/parameters	1070/4/41
*R*_1_ [*I* > 2*σ*(*I*)]	0.0317
w*R*_2_ (all data)	0.0876
Goodness of fit	1.020

**Table:** 

Atom	Site	*x*	*y*	*z*	Occupancy
La	4*e*	0.79764(4)	0.05501(4)	0.69670(4)	1
Ag(1)	4*e*	0.6767(9)	0.5949(4)	0.6353(9)	0.76(3)
Ag(2)	4*e*	0.6458(14)	0.6013(12)	0.6024(14)	0.24(3)
S(1)	4*e*	0.57065(18)	0.35088(19)	0.81673(19)	1
S(2)	4*e*	0.93082(18)	0.73793(19)	0.48968(18)	1

**Table:** 

Atom	*U*_11_ (Å^2^)	*U*_22_ (Å^2^)	*U*_33_ (Å^2^)	*U*_12_ (Å^2^)	*U*_13_ (Å^2^)	*U*_23_ (Å^2^)
La	0.00497(19)	0.0046(2)	0.00510(19)	−0.00026(10)	0.00068(13)	−0.00012(10)
Ag(1)	0.0114(10)	0.0085(3)	0.0188(11)	−0.0002(5)	0.0084(9)	0.0025(5)
Ag(2)	0.0114(10)	0.0085(3)	0.0188(11)	−0.0002(5)	0.0084(9)	0.0025(5)
S(1)	0.0059(6)	0.0072(6)	0.0067(5)	0.0011(5)	0.0008(4)	−0.0007(5)
S(2)	0.0058(6)	0.0064(6)	0.0057(6)	0.0002(5)	0.0017(4)	−0.0003(5)

**Table:** 

Bond	Length (Å)	Bond	Length (Å)
La–S(1)	2.9206(14)	Ag(1)–S(1)	2.416(3)
La–S(1)	2.9618(13)	Ag(1)–S(1)	2.622(4)
La–S(1)	2.9270(14)	Ag(1)–S(2)	2.475(3)
La–S(2)	2.9595(14)	Ag(1)–S(2)	3.031(6)
La–S(2)	3.0069(13)	Ag(2)–S(1)	2 × 2.517(9)
La–S(2)	2.9388(13)	Ag(2)–S(2)	2.512(8)
La–S(2)	3.0066(14)	Ag(2)–S(2)	3.082(10)

### Electronic structure calculations

Electronic structure calculations were performed using *ab initio* density functional theory (DFT) with the plane-wave pseudopotential method implemented in the CASTEP code.^51^ The Perdew–Burke–Ernzerhof (PBE)^52^ exchange–correlation functional with a plane-wave cutoff energy of 1150 eV was used. The *k*-point spacing was decided by a Monkhorst–Pack grid^53^ to give convergence of the total energy to a tolerance of ≤1 meV per atom. Geometry optimisations were converged to tolerances of: total energy (2 × 10^−5^ eV per atom), maximum ionic force (5 × 10^−2^ eV Å^−1^), maximum ionic displacement (1 × 10^−3^ Å), and maximum stress component (0.1 GPa). Calculations for LaAgS_2_ were carried out assuming a fully ordered model with Ag^+^ ions fully occupying a single 4*e* site.

## Results

### Lanthanum disulfide LaS_2_

The layered precursor LaS_2_ was synthesised by a high temperature solid state reaction as described previously.^22,23^ Reaction at 750 °C gave a mix of ∼32.7(4)% by weight of α-LaS_2_, 27.9(5)% by weight of β-LaS_2_ and unreacted sulfur (see SI). LaS_2_ was also prepared by ball milling La_2_S_3_ and S (in N_2_(g) atmosphere) to give a sample of LaS_2_ (probably a mix of α and β polymorphs) and a trace of La_3_S_4_ (Fig. 2a). Annealing this sample at 800 °C gave a sample with some sharper diffraction peaks, Fig. 2b, which was fitted by a stoichiometric phase of α-LaS_2_ with some broadening of *hk*0 reflections.^54^

**Fig. 2:**
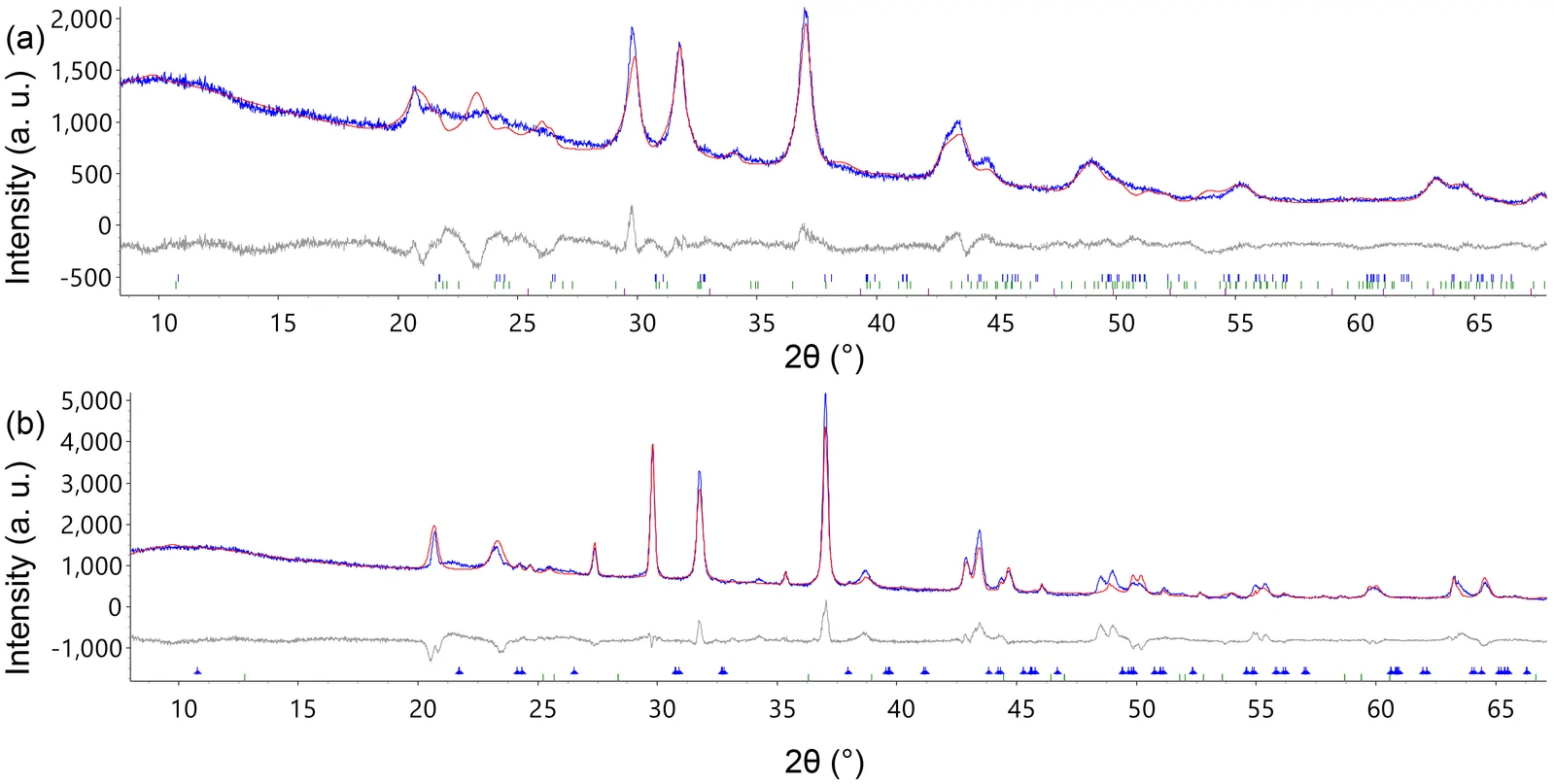
Rietveld refinement profiles for the synthesis of LaS_2_ from ball milling La_2_S_3_(s) with S(s) in N_2_(g) atmosphere with observed, calculated and difference profiles in blue, red and grey, respectively. Panel (a) shows sample immediately after ball milling with top (blue), middle (green) and bottom (purple) tick marks showing peak positions for α-LaS_2_ (42(1)% by weight), β-LaS_2_ (46(1)% by weight) and La_3_S_4_ (12(1)% by weight) respectively; *R*_wp_ = 6.50%, *R*_p_ = 4.97%, *χ*^2^ = 3.1. Panel (b) shows ball milled sample after annealing with top (blue), bottom (green) tick marks showing peak positions for α-LaS_2_ (94.3(2)% by weight) and La_2_O_2_S (5.7(2)% by weight) respectively; *R*_wp_ = 10.57%, *R*_p_ = 7.03%, *χ*^2^ = 8.1. X-ray wavelength is Cu Kα, 1.5418 Å.

### Lanthanum copper disulfide LaCuS_2_

High temperature solid state reactions can be used to synthesise LaCuS_2_^36,37,55^ and our attempts at high temperature synthesis produced the desired phase in the presence of La_2_S_3_ and other impurity phases (see SI). We were also able to synthesise LaCuS_2_ by mechanochemical topochemical (MT) reaction: LaS_2_(s) (prepared by ball milling, see above) was ball milled with Cu(s) in a N_2_(g) atmosphere to produce LaCuS_2_(s) (∼98% by weight) with a trace of Cu(s) also detected by XRPD (Fig. 3a). Further work to optimise the milling process may lead to improved sample purity, although we note that the purity of the LaS_2_ precursor (and exposure to air, *e.g.* during transfers in and out of the glovebox) may have limited our MT LaCuS_2_ sample purity. Similarly, a lower purity sample of LaCuS_2_ was also prepared by a low temperature topochemical (LTT) reaction: LaS_2_(s) was reacted with Cu(s) (see Methods section) to produce LaCuS_2_(s) (∼70% by weight) with some unreacted α-LaS_2_ and Cu(s) (Fig. 3b). Pelletising the reaction mixture, and further heating may improve sample purity. The model refined for LaCuS_2_ was consistent with the monoclinic (*P*2_1_/*c*) structure reported by Strobel *et al.*^38^ and Julien-Pouzol and Guittard.^55^

**Fig. 3:**
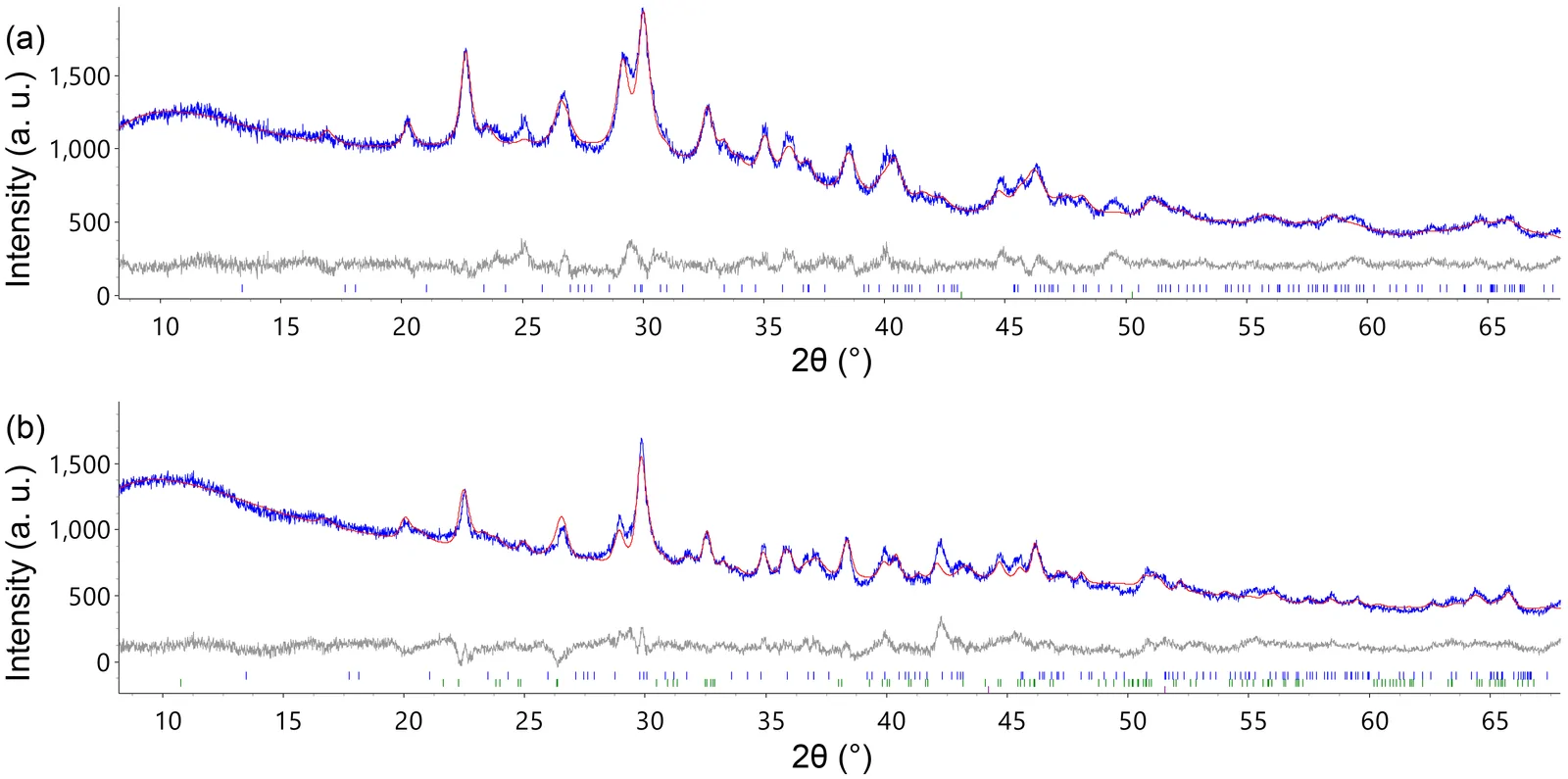
Rietveld refinement profiles for the synthesis of LaCuS_2_ with observed, calculated and difference profiles in blue, red and grey, respectively. Panel (a) shows sample prepared by ball milling LaS_2_(s) with Cu(s) in a N_2_(g) atmosphere, upper (blue) and lower (green) tick marks show peak positions for LaCuS_2_ (98(1)% by weight) and Cu (2.0(1)% by weight), respectively, *R*_wp_ = 3.86%, *R*_p_ = 3.00%, *χ*^2^ = 1.3. Panel (b) shows sample prepared by low temperature topochemical (LTT) reaction between LaS_2_(s) with Cu(s) at 400 °C, upper (blue), middle (green) and lower (purple) tick marks show peak positions for LaCuS_2_ (70.0(9)% by weight), α-LaS_2_ (21.6(9)% by weight) and Cu (8.4(5)% by weight), respectively, *R*_wp_ = 4.79%, *R*_p_ = 3.59%, *χ*^2^ = 1.8. X-ray wavelength is Cu Kα, 1.5418 Å.

Diffuse reflectance measurements on LaCuS_2_ prepared by MT reaction showed an increase in absorption for energy *E* ≥ ∼2 eV and a Tauc plot^56^ (Fig. 4a), assuming a direct band gap,^57^ indicated an optical band gap of ∼2.1(2) eV, consistent with its dark red-brown colour and similar to values measured for La_1−*x*_Y_*x*_CuS_2_.^58^

**Fig. 4:**
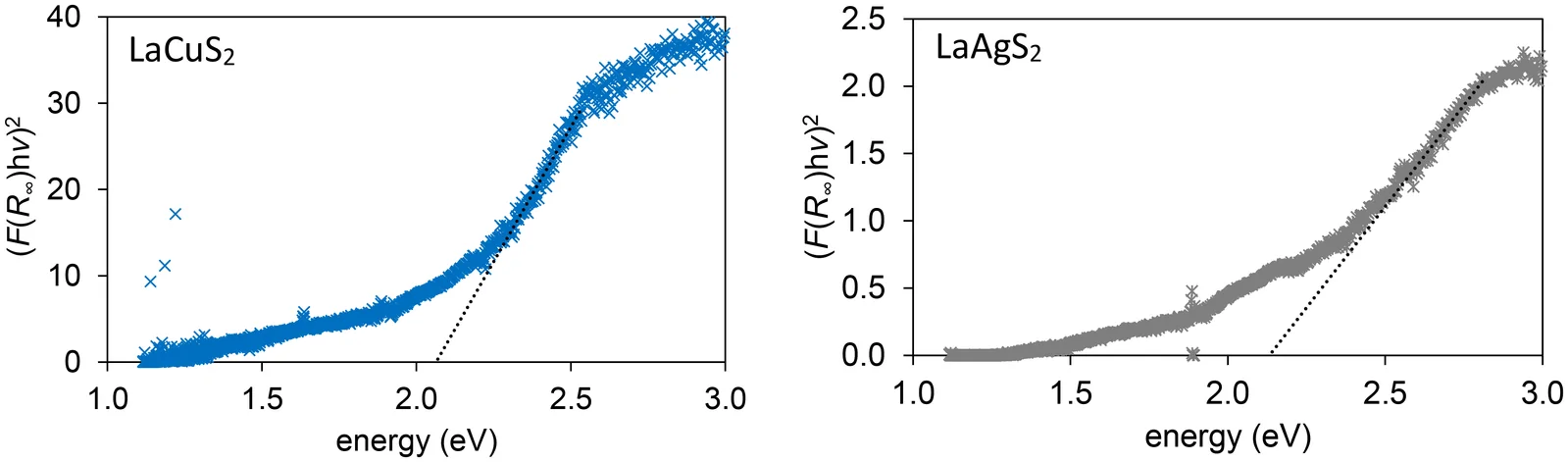
Tauc plots from diffuse reflectance measurements for (a) LaCuS_2_ and (b) LaAgS_2_ giving estimated optical direct band gaps of ∼2.1(2) eV for both materials.

### Lanthanum silver disulfide LaAgS_2_

To the best of our knowledge, LaAgS_2_ has not yet been reported, and our attempts to synthesise LaAgS_2_ by high temperature reaction between La_2_S_3_ and Ag_2_S were not very successful leading to multiphase samples (see SI). We therefore attempted LTT reactions to synthesise LaAgS_2_: XRPD data (Fig. 5a) indicated that a phase with a similar unit cell to LaCuS_2_ was observed for reactions between LaS_2_ and Ag at 300–500 °C. The relatively low reaction temperature results in samples that are not well annealed and have broad peaks in the XRPD data. The amount of unreacted Ag in the reaction mixture (with peak in XRPD data at ∼37° 2*θ*) decreases with increasing reaction temperature, but otherwise the XRPD patterns change very little with increasing reaction temperature. EDX analysis (SI) indicate a fairly homogeneous distribution of La, Ag and S (close to the expected ratios) for much of the sample, but with some particles of unreacted Ag. Introducing further heating steps or longer reaction times, and pelletising the reaction mixture, may improve sample purity, as long as care is taken to prevent air-exposure of the reagents. Rietveld refinement (Fig. 5b) using these data was consistent with the monoclinic (*P*2_1_/*c*) structure reported for LaCuS_2_ by Strobel *et al.*^38^ and Julien-Pouzol and Guittard.^55^

**Fig. 5:**
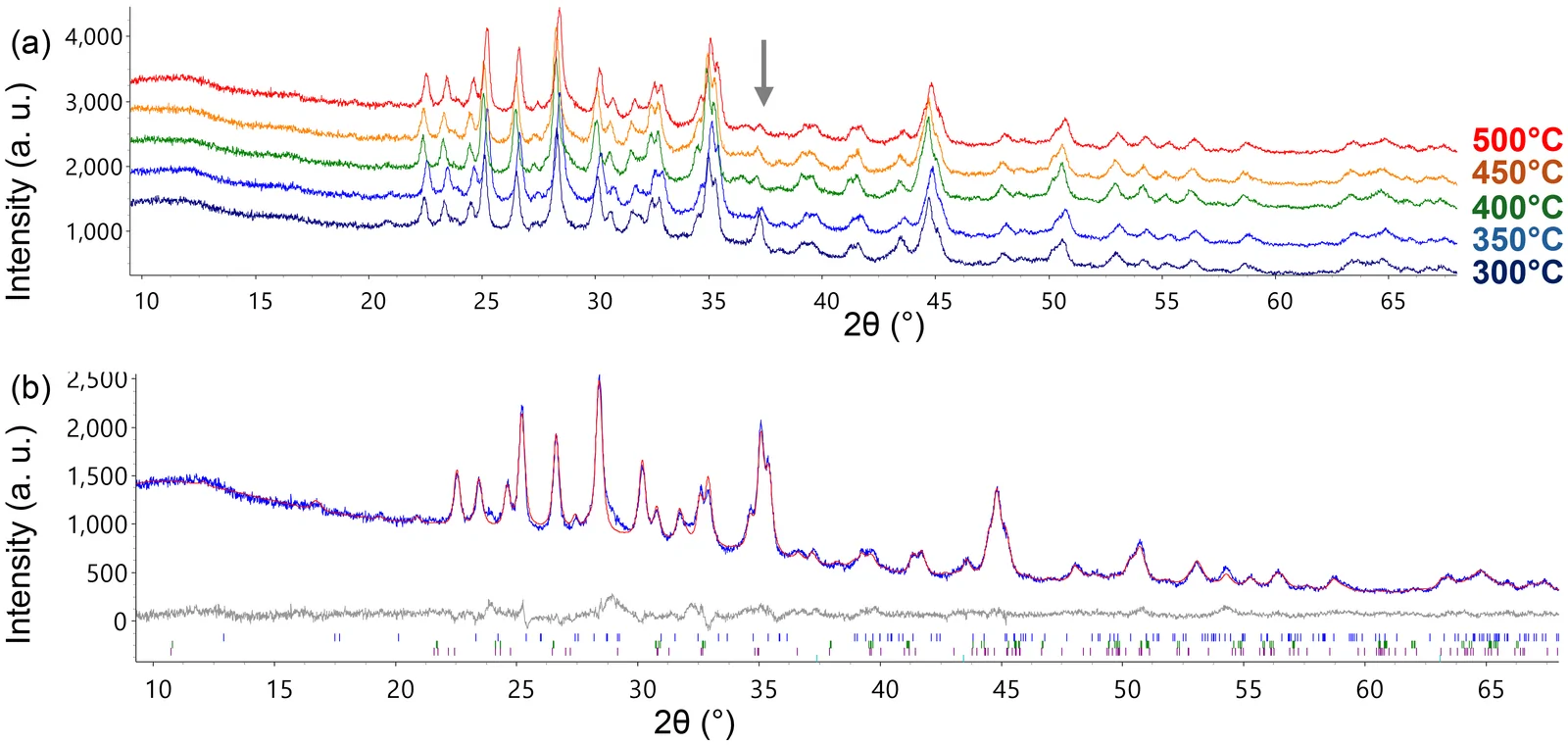
(a) Shows XRPD data collected for samples produced by low temperature topochemical (LTT) reactions between LaS_2_ and Ag with grey arrow indicating approximate position of the most intense peak from the Ag phase (note these data were collected in an air-sensitive sample holder hence the sample height varies between samples which is accounted for in later Rietveld refinements). Panel (b) shows Rietveld refinement profiles for data collected from LTT reaction to form LaAgS_2_ at 500 °C with observed, calculated and difference profiles in blue, red and grey, respectively; *R*_wp_ = 4.07%, *R*_p_ = 3.07%, *χ*^2^ = 1.4. Top (blue), upper middle (green), lower middle (purple) and bottom (cyan) tick marks show peak positions for LaAgS_2_ (94.4(2)% by weight), α-LaS_2_ (4.4(2)% by weight), β-LaS_2_ (0.4(1)% by weight) and Ag (0.91(6)% by weight). Refinement details are given in SI. X-ray wavelength is Cu Kα, 1.5418 Å.

The low reaction temperature is not sufficient to anneal the sample and so peaks in XRPD data are broadened (due to the small particle size), and these data are not suitable to confirm the crystal structure of this new material. Therefore single crystals of LaAgS_2_, in the form of black platelets, were grown from LaAgS_2_ powder prepared by LTT reactions in a CsCl flux, similar to the crystal growth reported for LaCuS_2_.^38^ These crystals were found to be air-stable up to at least 800 °C. Analysis of single crystal XRD data revealed the crystal to be a three-component twin, but a structural model (Fig. 6 and Table 1, with atomic displacement parameters constrained to be equal for the two partially-occupied Ag sites to reduce correlations with site occupancies) was determined and refined. (It was found to be consistent with that refined from XRPD data.) This single crystal XRD analysis allowed the crystal structure to be investigated more carefully which suggested some disorder of the Ag^+^ cations over two closely spaced 4*e* sites.

**Fig. 6:**
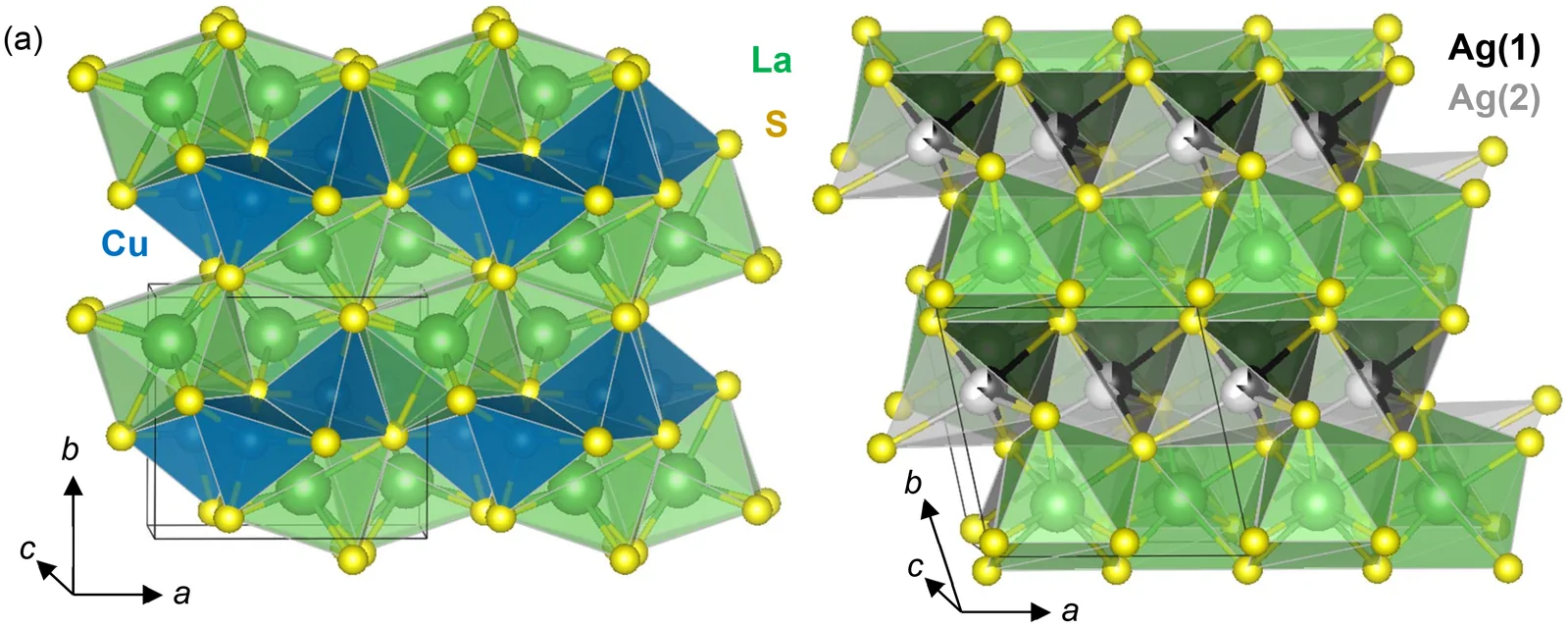
Illustration of crystal structures of (a) LaCuS_2_ and (b) LaAgS_2_ prepared here; La, Cu, Ag(1), Ag(2) and S sites are shown in green, blue, black, grey and yellow, with LaS_7_, CuS_4_ and AgS_5_ polyhedra are shown in green, blue, and grey, respectively.

Diffuse reflectance measurements on LaAgS_2_ prepared by LTT reaction showed a similar spectrum to LaCuS_2_ and again, a Tauc plot^56^ (Fig. 4b) assuming a direct band gap^57^ indicated an optical band gap of ∼2.1(2) eV.

### Electronic structure calculations

Density functional theory (DFT) was used to calculate formation energies for LaCuS_2_ and LaAgS_2_ from both binary sulfide reagents (reaction 1, akin to a high temperature reaction route), and from LaS_2_ and the metal (reaction 2, akin to a topochemical reaction involving anionic redox), Fig. 7. These calculations suggest that LaCuS_2_ is the thermodynamic product, consistent with its synthesis by high temperature reaction, and by low-temperature and mechanochemical topochemical reactions (Fig. 3). Reactions 1 and 2 to form LaAgS_2_ are only marginally enthalpically favourable, reaction 2 slightly more so, consistent with the greater success of our topochemical synthesis reactions (akin to reaction 2) compared with the high temperature reaction (more akin to reaction 1). The greater challenge in preparing this monoclinic phase of LaAgS_2_ compared with the copper analogue presumably results from the difficulty of introducing the relatively large Ag^+^ ion into a small site; indeed the only other ternary La–Ag–S phase reported,^35^ AgLa_5_S_8_, has Ag^+^ and La^3+^ ions disordered over 8-coordinate sites (with Ag–S bond distances of ∼3.01 Å).^59^ This results in LaAgS_2_ being less stable than LaCuS_2_ (calculated Δ*H*_f_ = −5.4 eV for LaAgS_2_ and −5.6 eV for LaCuS_2_).

**Fig. 7:**
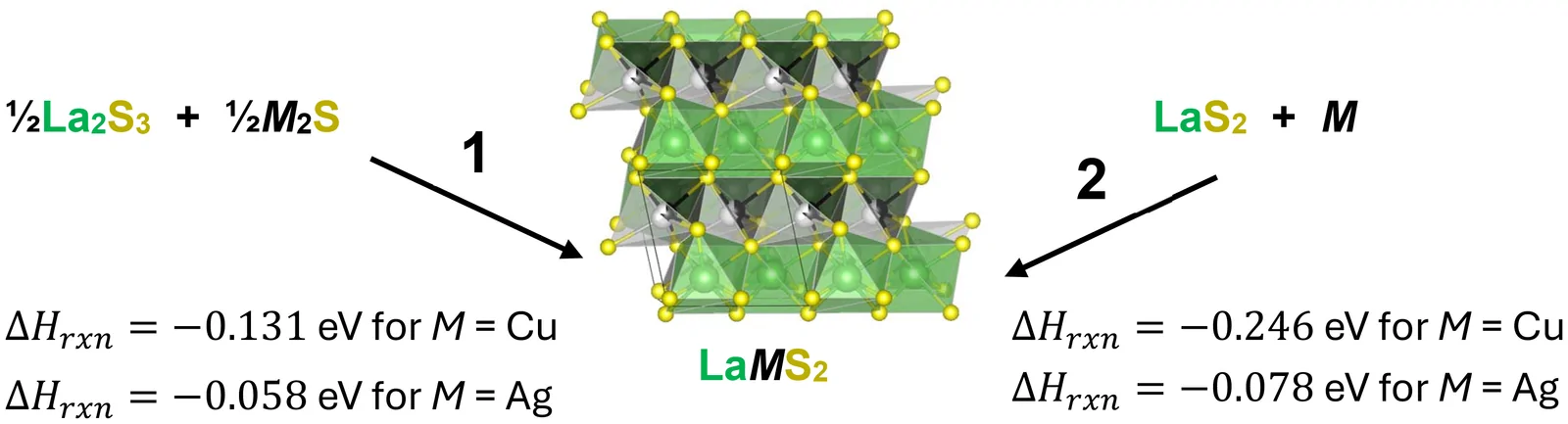
Scheme illustrating possible synthesis reactions to form La*M*S_2_ (*M* = Cu, Ag): reaction 1 is the high temperature solid state reaction, and reaction 2 is the topochemical reaction involving anionic redox.

The electronic density of states (Fig. 8) reveals a clear difference between LaCuS_2_ and LaAgS_2_ with the latter having a larger calculated band gap (1.57 eV) than LaCuS_2_ (1.07 eV). Although both values are smaller than the experimentally determined optical band gaps (Fig. 4), this behaviour is entirely consistent with the well-known tendency of semi-local PBE density functional theory to underestimate band gaps.^52^ The calculations nevertheless reproduce the correct qualitative trend across the series, indicating that replacing Cu by Ag increases the band gap. A more quantitative description could be obtained using a higher-rung exchange–correlation functional, such as rSCAN^60^ or a screened hybrid functional (*e.g.* HSE06),^61^ although these approaches are computationally more demanding. The projected density of states shows that the valence-band maximum is dominated by hybridised S 3p and Cu 3d states and the conduction-band minimum is primarily La 5d in character. The larger band gap in LaAgS_2_ therefore reflects the modified interaction between the Ag 4d and S 3p states.

**Fig. 8:**
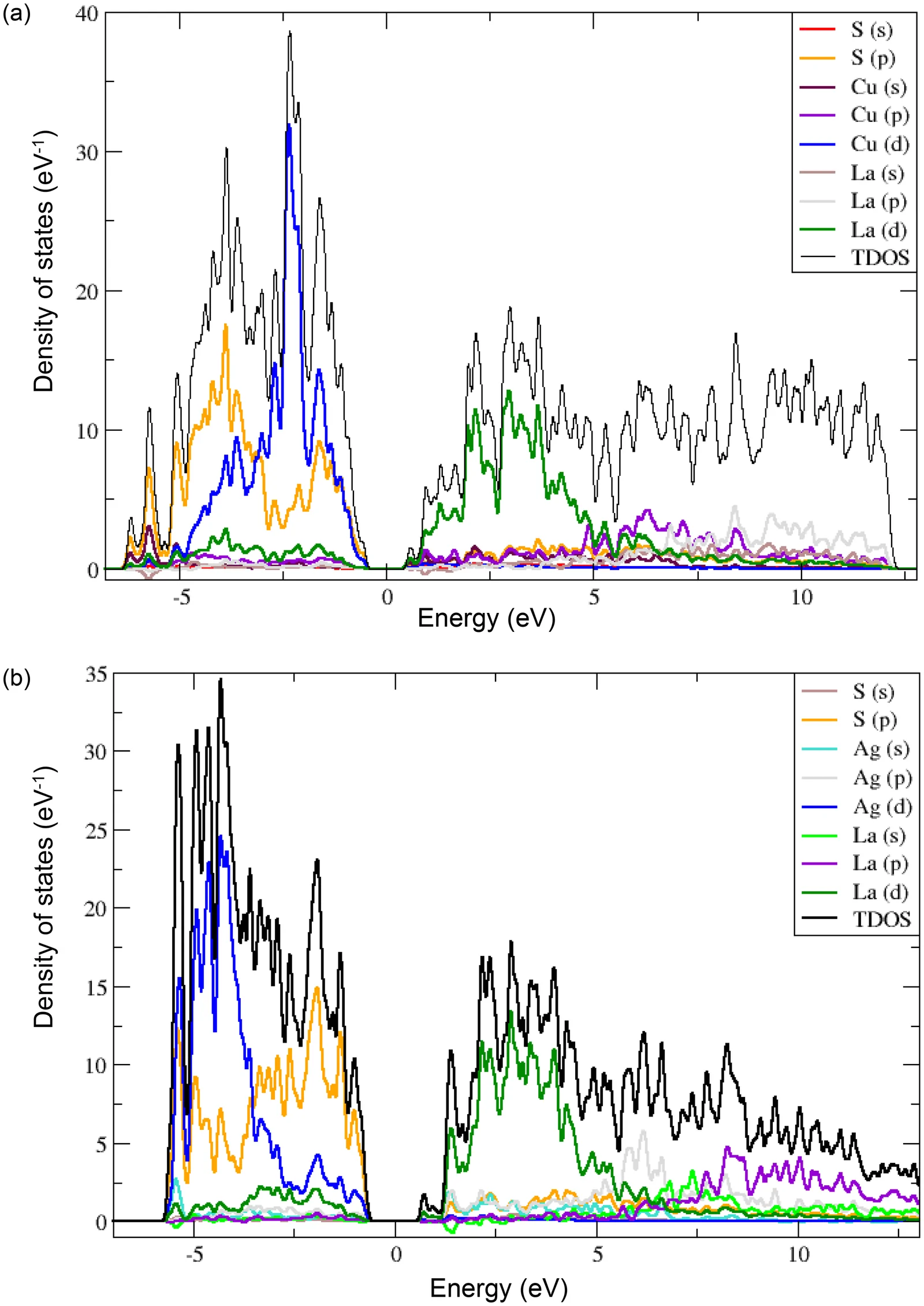
Projected density of states for (a) LaCuS_2_ and (b) LaAgS_2_. Calculations were carried out using PBE. It should be noted that PBE tends to underestimate bandgap values in semiconductors.

## Discussion

Our study focuses on the synthesis of ternary sulfides La*M*S_2_ (*M* = Cu, Ag) using the precursor LaS_2_. LaS_2_ was first prepared by high temperature solid state reactions^22–24^ but research into synthesis methods for this optoelectronic material are ongoing^62^ with reports of synthesis by metathesis reactions,^25,63^ microwave-assisted metathesis reactions,^64^ hydrothermal methods,^65^ and most recently, as colloidal nanocrystals from LaI_3_ in oleylamine.^66^ We can add our synthesis by ball milling to this list. It would be interesting to explore whether this route could be applied to other lanthanide dichalcogenides.^67^ Annealing our ball-milled samples gave a diffraction pattern with some sharp diffraction peaks, alongside other more broad peaks. It would be interesting to explore, perhaps using electron microscopy, the microstructure of the sample and the ratio of α and β polymorphs in the sample.

LaCuS_2_ has typically been synthesised by high temperature solid state reactions (*T* ≥ 800 °C),^36–38^ although recently LaCuS_2_ has also been prepared by a sol–gel route for photocatalysis applications.^68^ Sasaki *et al.* demonstrated that the ternary selenide LaCuSe_2_ could be prepared by LTT reaction of LaSe_2_ with Cu,^13^ and it is pleasing to extend this method to the ternary sulfide LaCuS_2_ in this work. To date, these anionic redox topochemical reactions are typically carried out by heating the precursor and transition metal element to low temperatures in an evacuated, sealed, quartz tube^13,21^ but Sasaki *et al.* have shown that analogous reactions can be performed by ball milling,^14^ as we have demonstrated here to prepare LaCuS_2_ (Fig. 3a).

Our synthesis of LaAgS_2_ by this LTT method (Fig. 5) highlights the importance of topochemical anionic redox reactions for preparing metastable phases.^15,19,20^ To the best of our knowledge, LaAgS_2_ has not been reported, and our attempts to synthesise it by conventional high temperature solid state reactions gave mixed-phase samples containing the possibility of only ∼9.5(2)% (by weight) of LaAgS_2_. However, several *Ln*AgS_2_ phases with smaller lanthanide cations are known, including those of AgGdS_2_-type structure (*P*2_1_ symmetry), AgYbS_2_-type structure (*I*4_1_*md* or *I*4_1_/*amd* symmetry), and NaCl-type structure (*Fm*3̄*m* symmetry), as summarised in Fig. 9. Unlike the *Ln*CuS_2_ structures built from corner and-/or edge-linked CuS_4_ tetrahedra (see ref. 32–35 SI and LaCuS_2_ illustrated in Fig. 6a), the reported *Ln*AgS_2_ structures, containing the larger Ag^+^ ions (4-coordinate ionic radii of Cu^+^ and Ag^+^ are 0.60 Å and 1.00 Å, respectively),^69^ are composed of 6-coordinate *Ln*^3+^ sites built around 5- or 6-coordinate Ag^+^ sites^40–42^ (Fig. 9). The anionic redox topochemical reaction can be considered in terms of a “zipper” mechanism with metals being oxidised to cations as they break apart the (S_2_)^2−^ dimers which get reduced.^15^ The layers of (S_2_)^2−^ dimers in the α and β polymorphs of LaS_2_ (Fig. 1) lend themselves to give 4-coordinate sites for the oxidised *M*^+^ ions (see LaCuS_2_ structure illustrated in Fig. 6a). Hence our LTT synthesis of LaAgS_2_, with the low reaction temperature likely allowing kinetics to play a role, results in a structure very similar to that of LaCuS_2_, despite the much larger size of the Ag^+^ ion. The *M*S_4_ sites in LaAgS_2_ are quite distorted, closer to elongated trigonal bipyramidal sites which are only corner-linked (and not edge-linked as in LaCuS_2_). The Ag^+^ ions are slightly displaced from the trigonal plane of these sites, moving towards the (approximately) tetrahedrally coordinated site occupied by Cu^+^, but being larger, Ag^+^ ions cannot occupy the smaller tetrahedral site. This splitting of the of Ag^+^ sites is similar to the Cu^+^ displacements described by the chalcohalide CuBiSeCl_2_ and could contribute to low thermal conductivity.^70^ The lower-symmetry coordination environment observed here for Ag^+^ is similar to that expected for ns^2^ lone pair cations such as Sn^2+^, Pb^2+^, Sb^3+^. We therefore attempted LTT reactions between Sn and LaS_2_ (assuming half-occupancy of the cation sites by divalent Sn^2+^ for charge balance) but these were not successful and instead resulted in S-deintercalation, consistent with other reported attempts at anionic redox reactions involving *M*^2+^ cations.^71,72^ It would be interesting to explore similar LTT reactions with other *M*^+^ ions; reaction with Tl^+^ might allow the influence of the second-order Jahn–Teller effect for ns^2^ ions to be investigated. The elongated Ag^+^ sites in LaAgS_2_ are accommodated by the greater monoclinic distortion (*β* ≫ 90° for LaAgS_2_) and flattening of the La–S layers compared with LaCuS_2_. It would be interesting to see whether *P*2_1_/*c* LaAgS_2_-like polymorphs could be prepared by similar topochemical reactions for other smaller lanthanides.

**Fig. 9:**
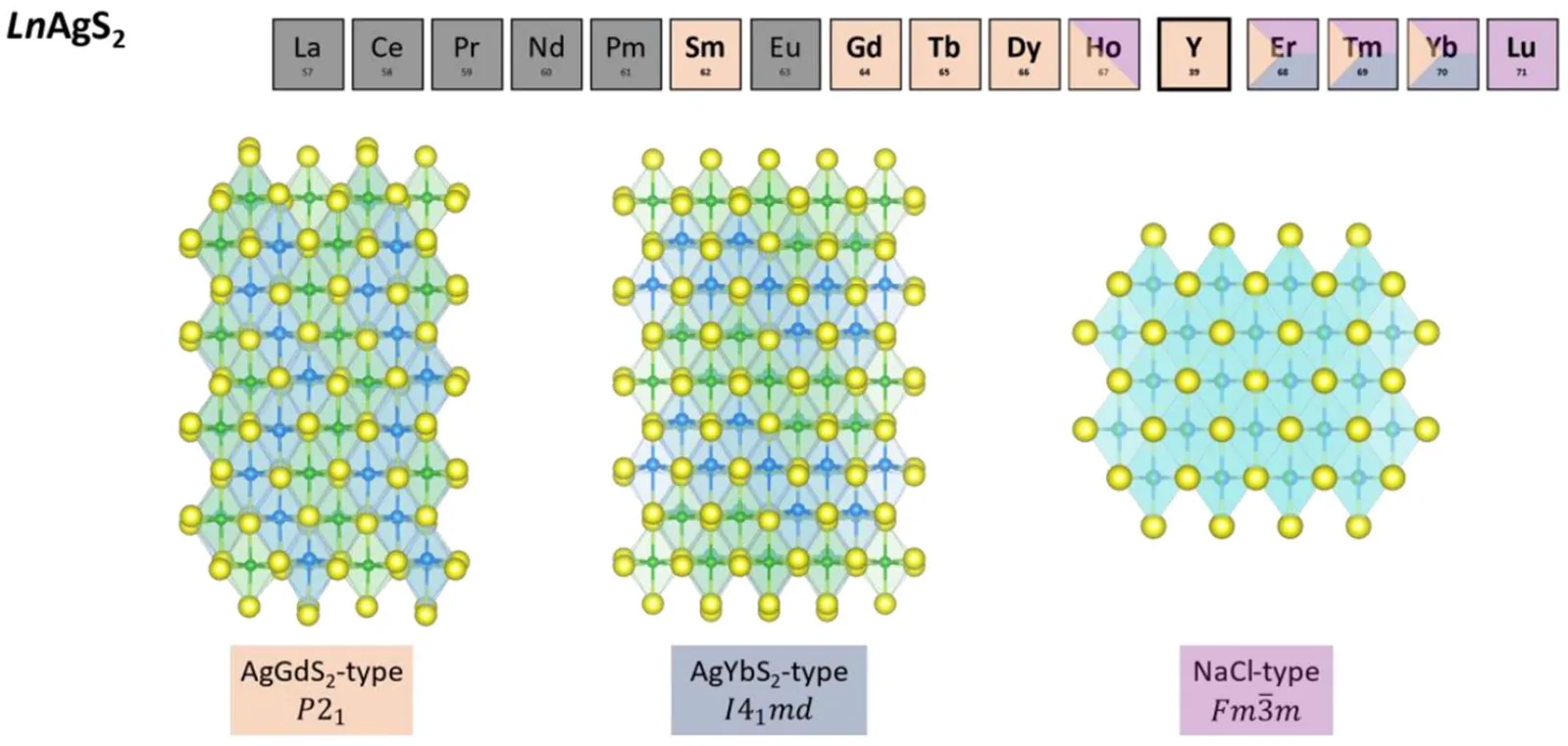
Schematic summarising the reported structure types for LnAgS_2_, with Ln cations coloured grey, orange, blue and pink indicated no structure report, AgGdS_2_ structure, AgYbS_2_ structure and NaCl structure, respectively.^35,40–42^ Ln, Ag and S ions are shown in green, blue and yellow, with Ln and Ag polyhedra in green and blue, respectively, and cation-disordered polyhedral in cyan. Y^3+^ has been included here due to its comparable ionic radius with Ho^3+^ and Er^3+^.^69^

## Conclusions

The transparent p-type semiconductor LaCuOS was first prepared by topochemical oxidation of LaCuS_2_^18^ and we report here the synthesis of this ternary sulfide LaCuS_2_ by low temperature topochemical reaction involving anionic redox, exploiting the reactive (S_2_)^2−^ dimers in the layered LaS_2_ precursor. This topochemical approach also enabled the synthesis of the new ternary sulfide LaAgS_2_. DFT calculations indicate that the synthesis of monoclinic LaAgS_2_ has relatively small thermodynamic stability and it is likely that kinetics play a role in this topochemical synthesis route. This results in a monoclinic structure for LaAgS_2_ which is closely related to that of LaCuS_2_, despite the larger size of the Ag^+^ cation. Diffuse reflectance measurements indicate that both materials are semiconductors with band gaps ∼2.1(2) eV, highlighting the potential of these materials for optoelectronic and photocatalytic applications. These findings highlight anionic redox topochemistry as a powerful strategy for preparing chalcogenides. It would be interesting to extend this work to explore whether new polymorphs of other ternary sulfides (including other *Ln*AgS_2_ phases with smaller lanthanide cations, and Bi-containing *A*Bi*Q*_2_ chalcogenides^6^) could be synthesised by similar anionic redox topochemical routes.

## Author contributions

Glen R. Hebberd: conceptualisation, investigation, formal analysis, data curation, writing – original draft. Toby J. Blundell: formal analysis. Stewart J. Clark: supervision, writing – review & editing. Leon Bowen: investigation. Emma E. McCabe: supervision, funding acquisition, data analysis, tube sealing, writing – review & editing.

## Conflicts of interest

There are no conflicts to declare.

## Supplementary Material

DT-055-D6DT02059C-s001

## Data Availability

The data supporting this article have been included as part of the supplementary information (SI) as appropriate. Supplementary information is available. See DOI: https://doi.org/10.1039/d6dt02059c. CCDC 2580779 (LaAgS_2_) contains the supplementary crystallographic data for this paper.^73^
