# Supplementary material for: Topochemical synthesis of ternary metal sulfides including new LaAgS2
Source: Dalton Trans. 2026 Sep 30;55(40):14601–13. doi: 10.1039/d6dt02059c (PMC13639657; doi:10.1039/d6dt02059c)
Supplement: Supplementary file 1 [file DT-055-D6DT02059C-s001.pdf]

Supplementary Information (SI) for Dalton Transactions.
This journal is © The Royal Society of Chemistry

Topochemical synthesis of ternary metal sulfides including new $LaAgS_2$

Glen R. Hebberd,[1] Toby J. Blundell,[2] Leon M. Bowen,[1] Stewart J. Clark,[1] Emma E. McCabe[1]*

[1] Department of Physics, Durham University, South Road, Durham, DH1 3LE, U. K.

[2] Department of Chemistry, Durham University, South Road, Durham, DH1 3LE, U. K.

Supporting information

SI1 Summary of $LnCuS_2$ structures

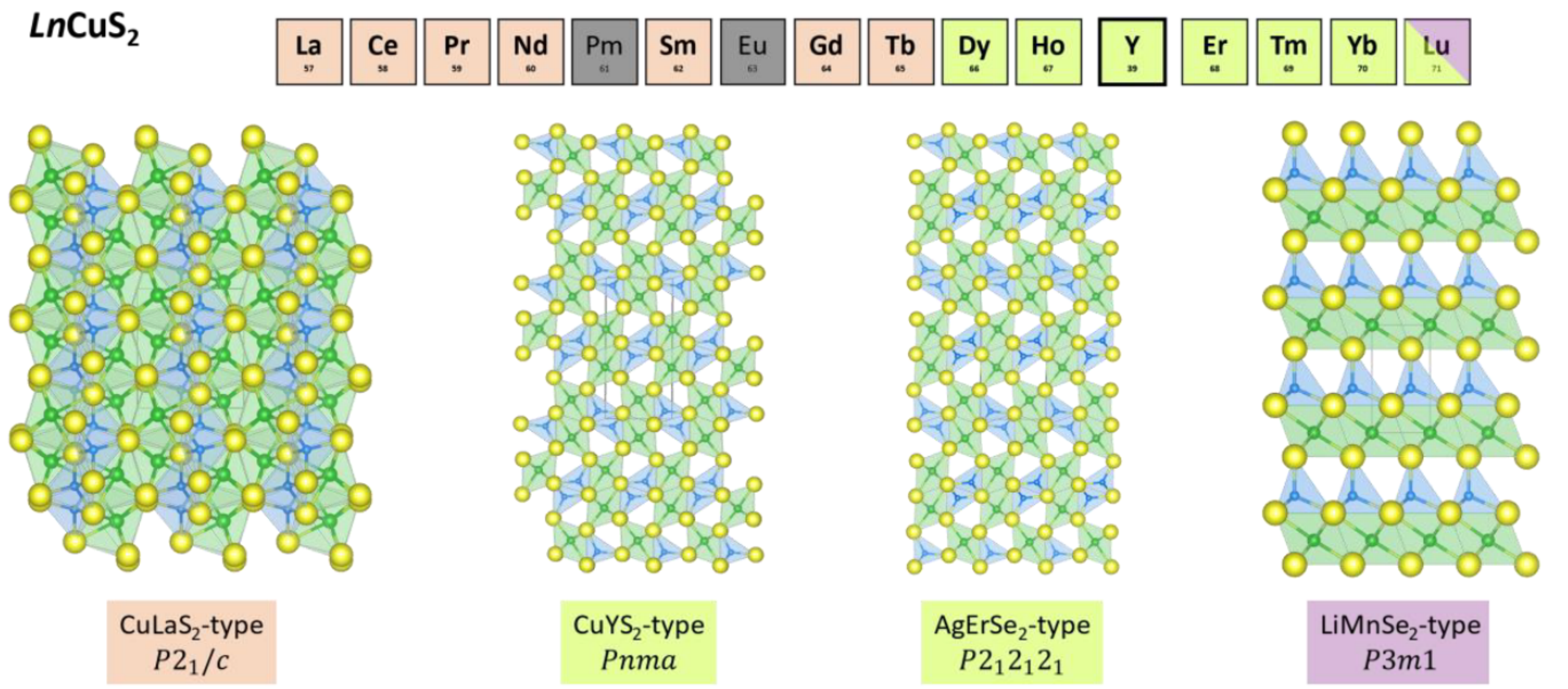

Figure SI1 A summary of the structure types reported for $LnCuS_2$ phases, as given in the ICSD.[1] The *Ln* species are colour-coded based on the structure type the $LnCuS_2$ composition exhibits, with Y slotted between Ho and Er in the series due to its ionic radii.[2] The *Ln* species are represented in green, with Cu in blue, and S in yellow. Green and blue polyhedra are displayed to show the coordination of *Ln* and Cu, respectively.

SI2 Synthesis of Cu_2S and Ag_2S precursors

Solid state reaction of 2Cu(s) + S(s) → Cu_2S(2) at 600°C

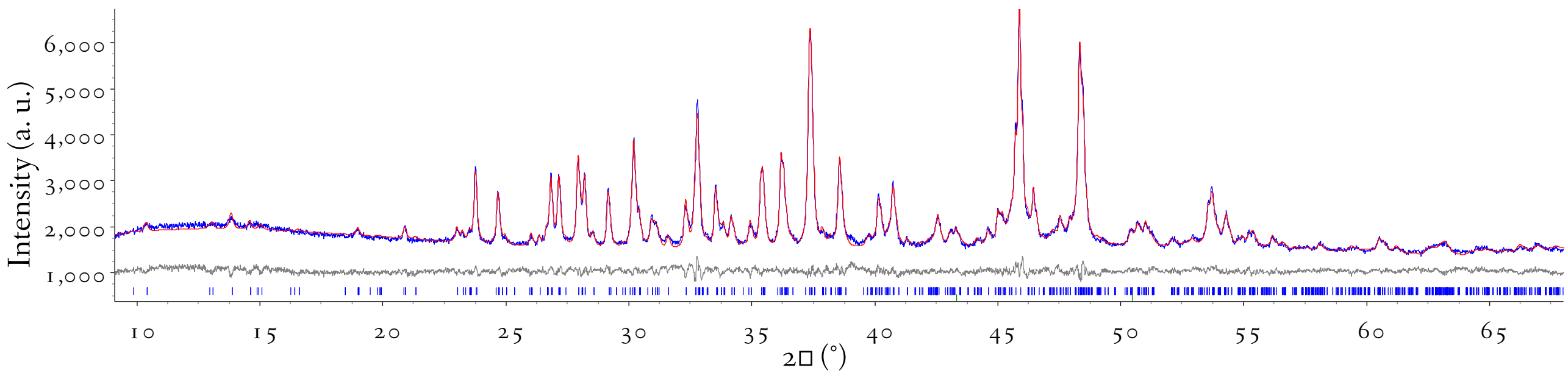

Figure SI2 Rietveld refinement profiles for synthesis of precursor Cu_2S showing observed, calculated and difference profiles in blue, red and grey, respectively; R_{wp} = 2.73%, R_p = 2.17%, χ^2 = 1.4. Top (blue) and bottom (green) tick marks show peak positions for Cu_2S (99.41(3)% by weight) and Cu (0.59(3)% by weight). X ray wavelength is Cu Kα, 1.5418 Å.

Solid state reaction of 2Ag(s) + S(s) → Ag_2S(2) at 250°C

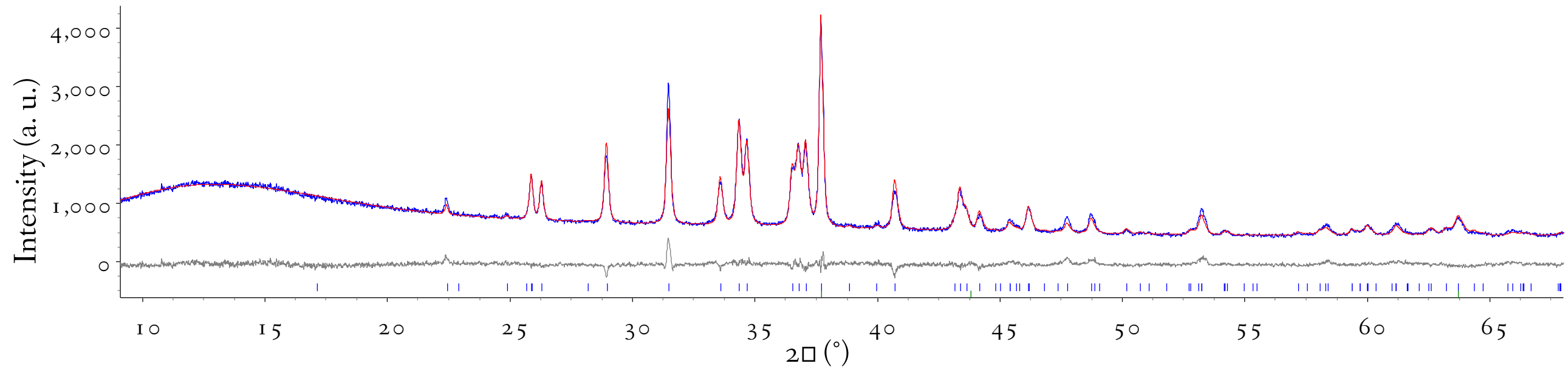

Figure SI3 Rietveld refinement profiles for synthesis of precursor Ag_2S showing observed, calculated and difference profiles in blue, red and grey, respectively; R_{wp} = 3.86%, R_p = 2.86%, χ² = 1.2. Top (blue) and bottom (green) tick marks show peak positions for Ag_2S and Al (diffractometer sample holder). X ray wavelength is Cu Kα, 1.5418 Å.

SI3 Synthesis of LaS_2

High temperature solid state reaction of La_2S_3(s) + S(s) → 2LaS_2(s) at 750°C

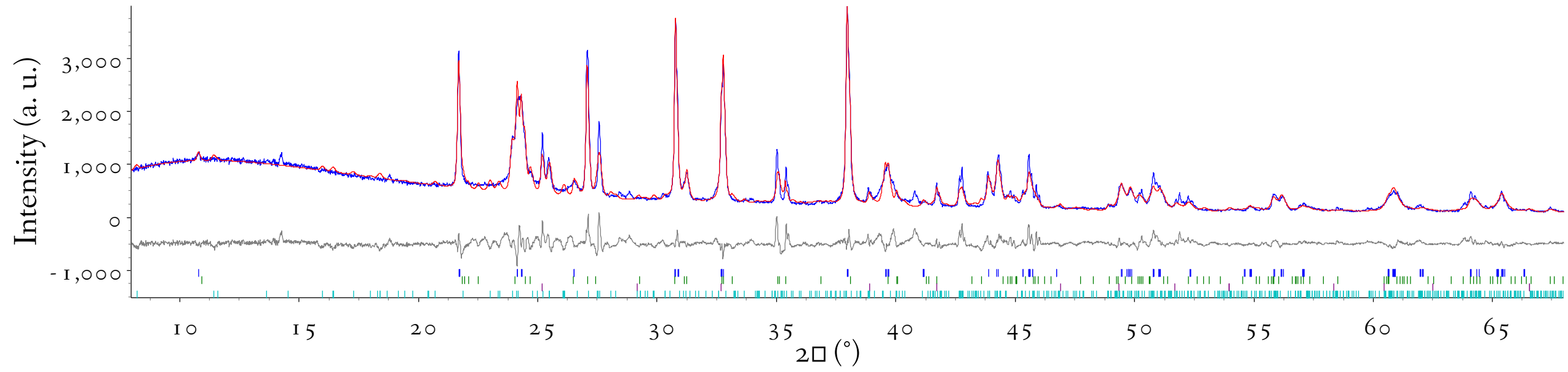

Figure SI4 Rietveld refinement profiles for high temperature solid state synthesis of LaS_2 showing observed, calculated and difference profiles in blue, red and grey, respectively; R_{wp} = 11.21%, R_p = 7.68%, χ² = 6.8. Top (blue), upper middle (green), lower middle (purple) and bottom (cyan) tick marks show peak positions for α-LaS_2 (32.7(4)% by weight), β-LaS_2 (27.9(5)% by weight), La3S4 (0.87(6)% by weight) and S (38.6(6)% by weight). X ray wavelength is Cu Kα, 1.5418 Å.

Table SI1 Refinement details from Rietveld refinement for ball milled LaS_2 sample after annealing; refinement profiles shown in Figure 2b of the main text. Refinements were carried out using a model of $P2_1/a$ symmetry (non-standard setting of space group 14, $P2_1/c$, for ease of comparison with other structure reports)[3,4], with a = 8.1657(7) Å, b = 4.1279(3) Å, c = 8.1881(7) Å, β = 90.02(2)°. A single global atomic displacement parameter was fixed for all phases.

Atom	Site	x	y	z	occupancy
La	*4e*	0.136(1)	0.779(1)	0.7121(5)	1
S(1)	*4e*	0.125(7)	0.768(6)	0.327(1)	1
S(2)	*4e*	0.112(4)	0.287(8)	1.004(2)	1

SI4 Synthesis of $LaCuS_2$

High temperature solid state reaction of La_2S_3(s) + Cu_2S(s) $\rightarrow$ 2$LaCuS_2$(s) at 900°C

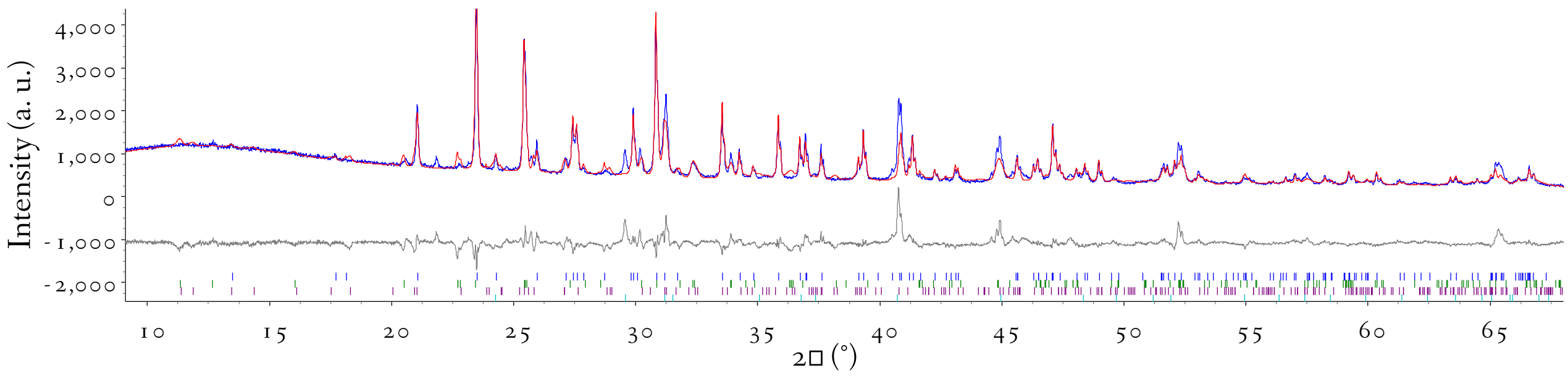

Figure SI5 Rietveld refinement profiles for high temperature solid state synthesis of $LaCuS_2$ showing observed, calculated and difference profiles in blue, red and grey, respectively; R_{wp} = 11.06%, R_p = 7.46%, χ^2 = 8.3. Top (blue), and upper middle (green), lower middle (purple) and bottom (cyan) tick marks show peak positions for $LaCuS_2$ (65.5(8)% by weight) and La_2S_3 (11.2(3)% by weight), $La_{10}OS_{14}$ (11.7(3)% by weight) and CuS_2 (12(1)% by weight), respectively. X ray wavelength is Cu Kα, 1.5418 Å.

SI5 Synthesis of $LaAgS_2$

High temperature solid state reaction of La_2S_3(s) + Ag_2S(s) $\rightarrow$ 2$LaAgS_2$(s) at 800°C

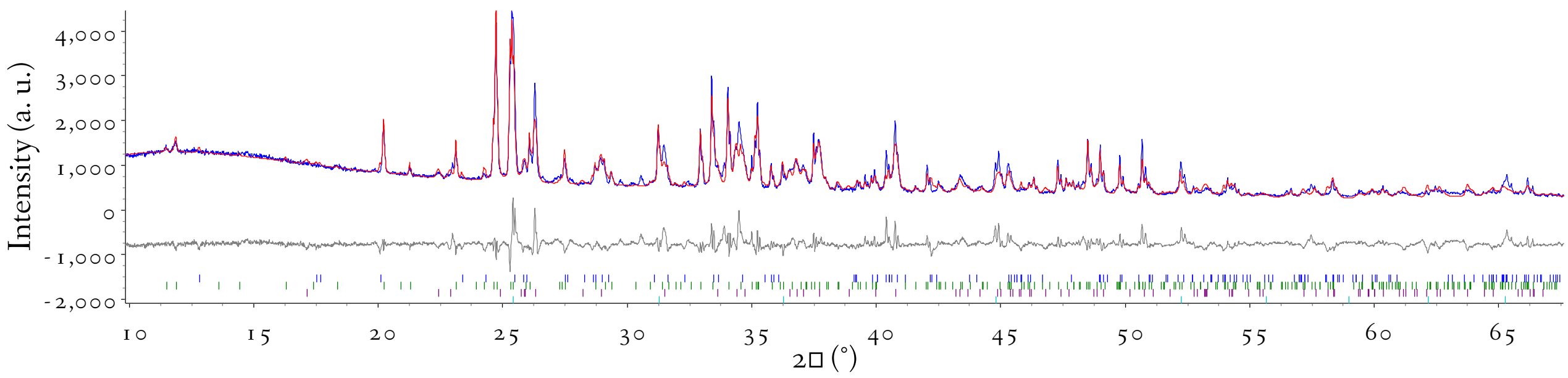

Figure SI6 Rietveld refinement profiles for high temperature solid state synthesis of $LaAgS_2$ showing observed, calculated and difference profiles in blue, red and grey, respectively; R_{wp} = 9.96%, R_p = 6.84%, χ^2 = 7.5. Top (blue), upper middle (green), lower middle (purple) and bottom (cyan) tick marks show peak positions for $LaAgS_2$ (9.5(2)% by weight), $La_{10}OS_{14}$ (51.7(3)% by weight), and Ag_2S (34.5(3)% by weight) and Ag_2O_3 (4.30(9)% by weight), respectively. X ray wavelength is Cu Kα, 1.5418 Å.

Low temperature solid state reaction of LaS_2(s) + Ag(s) $\rightarrow$ $LaAgS_2$(s) at 500°C

Energy-dispersive X-ray analysis (EDX) of $LaAgS_2$ samples prepared by LTT reaction at 500°C (Figure SI7) indicate that La, Ag and S are dispersed throughout the sample with molar ratios close to the expected values La:Ag:S of 25%:25%:50%, with additional particles of unreacted Ag present, consistent with analysis of XRPD data (Figure 5 of

the main text). The La content is slightly lower than the desired stoichiometry of 25%; this may be an artifact from the stronger X-ray absorption by heavier elements (particularly noticeable for thicker regions of each specimen).

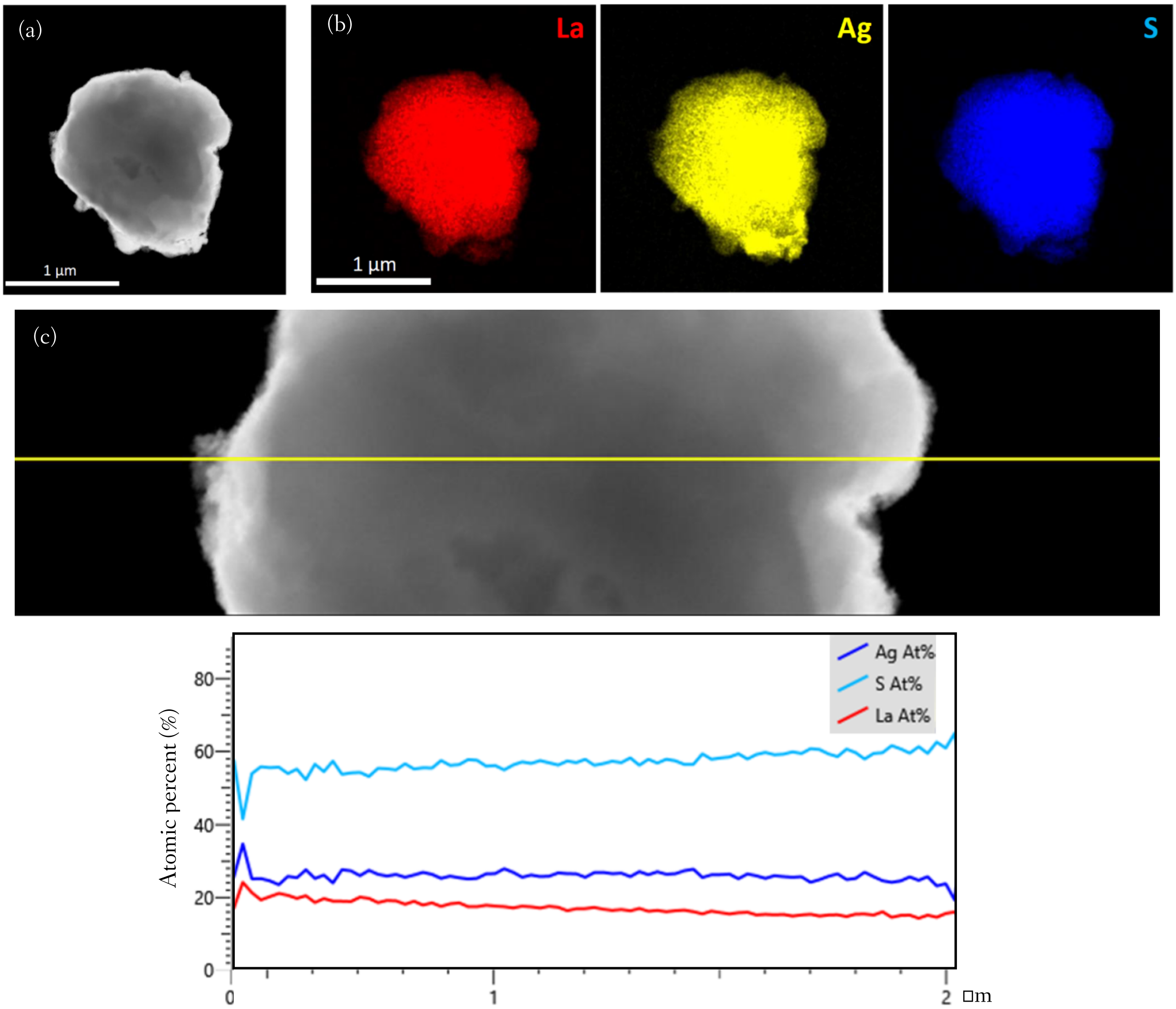

Figure SI7 EDX mapping of a particle of LTT $LaAgS_2$ sample synthesised at 500°C, showing (a) secondary electron image of the analysed area, (b) shows elemental maps with La (red), Ag (yellow), and S (blue); (c) shows a linescan through a section of the particle with the atomic percentages of each element as a function of position shown below.

References

(1) Zagorac, D.; Muller, H.; Ruehl, S.; Zagorac, J.; Rehme, S. Recent developments in the Inorganic Crystal Structure Database: theoretical crystal structure data and related features. *J. Appl. Cryst.* **2019**, *52* (5), 918-925. DOI: doi:10.1107/S160057671900997X.

(2) Shannon, R. D. Revised effective ionic radii and systematic studies of interatomic distances in halides and chalcogenides. *Acta Cryst. A* **1976**, *32* (SEP1), 751-767. DOI: 10.1107/s0567739476001551.

(3) Bartsch, C.; Doert, T. Ternary lanthanum sulfide selenides α-$LaS_{2-x}Se_x$ (0<x<2) with mixed dichalcogenide anions X_2^{2-} (X=S, Se). *J. Solid State Chem.* **2012**, *185*, 101-106. DOI: https://doi.org/10.1016/j.jssc.2011.10.039.

(4) Schleid, T.; Lauxmann, P.; Bartschb, C.; Doert, T. Lanthanoiddisulfide – Synthesen und Kristallstrukturen von α-CeS_2, α-NdS_2, β-LaS_2, β-CeS_2 und β-PrS_2 / Rare Earth Metal Disulfides – Syntheses and Crystal Structures of α-CeS_2, α-NdS_2, β -LaS_2, β -CeS_2, and β -PrS_2. *Z. Naturforsch. B* **2009**, *64* (2), 189-196. DOI: doi:10.1515/znb-2009-0208 (accessed 2026-07-24).
